# Supplementary material for: Association between oral microbiome and seven types of cancers in East Asian population: a two-sample Mendelian randomization analysis
Source: Front Mol Biosci. 2023 Nov 21;10:1327893. doi: 10.3389/fmolb.2023.1327893 (PMC10702768; doi:10.3389/fmolb.2023.1327893)
Supplement: Supplementary file 1 [file DataSheet1.ZIP › Supplementary Materials/MR plots for tongue/tongue═╝/Gastric cancer/pheno.3124_to_gastric cancer_leave_one_out.pdf]

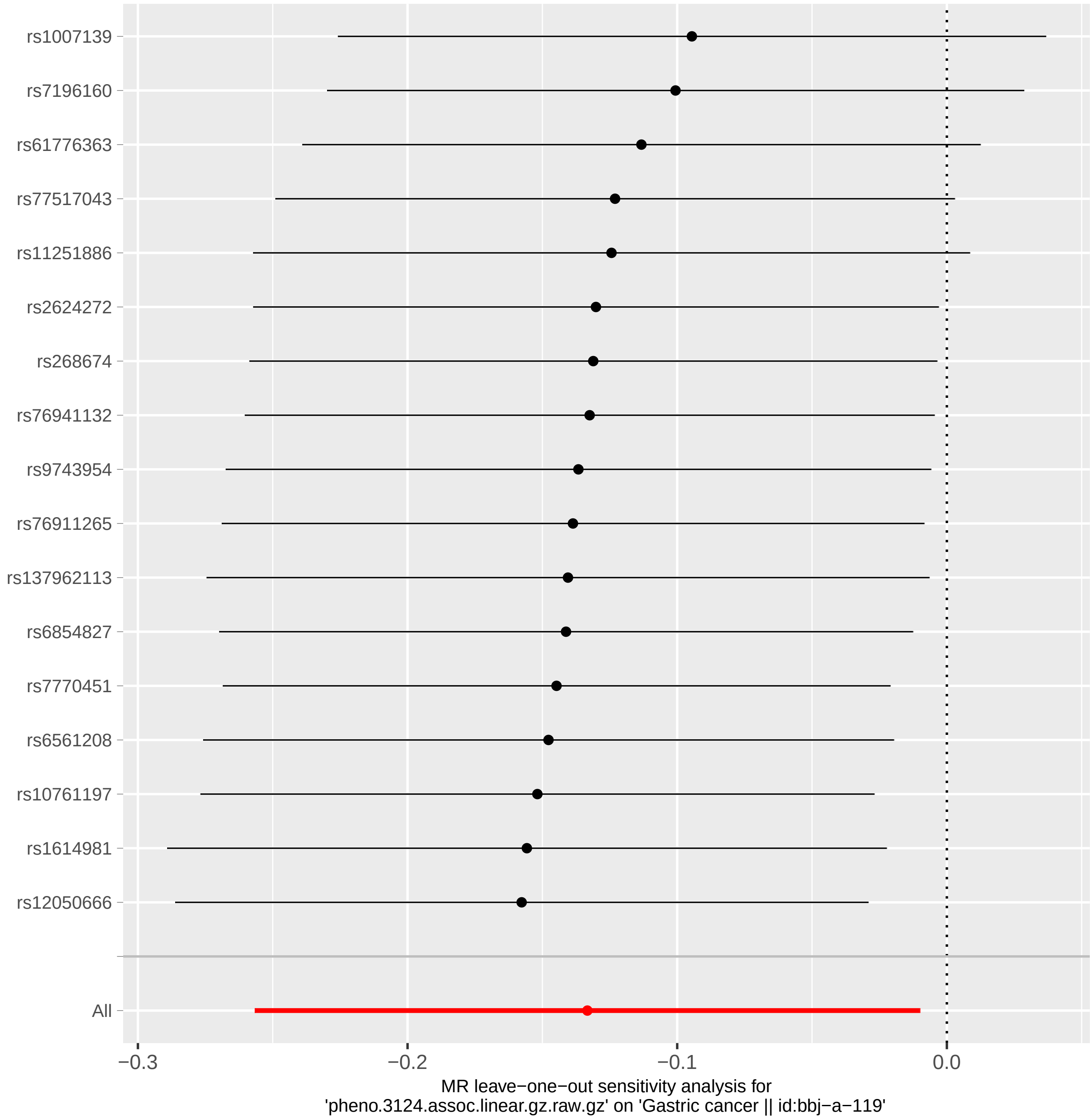
rs1007139
rs7196160
rs61776363
rs77517043
rs11251886
rs2624272
rs268674
rs76941132
rs9743954
rs76911265
rs137962113
rs6854827
rs7770451
rs6561208
rs10761197
rs1614981
rs12050666
All
−0.3
−0.2
−0.1
0.0
MR leave−one−out sensitivity analysis for
'pheno.3124.assoc.linear.gz.raw.gz' on 'Gastric cancer || id:bbj−a−119'
